# Erythroleukemia cells acquire an alternative mitophagy capability

**DOI:** 10.1038/srep24641

**Published:** 2016-04-19

**Authors:** Jian Wang, Yixuan Fang, Lili Yan, Na Yuan, Suping Zhang, Li Xu, Meilan Nie, Xiaoying Zhang, Jianrong Wang

**Affiliations:** 1 Hematology Center of Cyrus Tang Medical Institute, Jiangsu Institute of Hematology, Collaborative Innovation Center of Hematology, Jiangsu Key Laboratory for Stem Cell Research, Soochow University School of Medicine, Suzhou 215123, China

## Abstract

Leukemia cells are superior to hematopoietic cells with a normal differentiation potential in buffering cellular stresses, but the underlying mechanisms for this leukemic advantage are not fully understood. Using CRISPR/Cas9 deletion of the canonical autophagy-essential gene *Atg*7, we found that erythroleukemia K562 cells are armed with two sets of autophagic machinery. Alternative mitophagy is functional regardless of whether the canonical autophagic mechanism is intact or disrupted. Although canonical autophagy defects attenuated cell cycling, proliferation and differentiation potential, the leukemia cells retained their abilities for mitochondrial clearance and for maintaining low levels of reactive oxygen species (ROS) and apoptosis. Treatment with a specific inducer of mitophagy revealed that the canonical autophagy-defective erythroleukemia cells preserved a mitophagic response. Selective induction of mitophagy was associated with the upregulation and localization of RAB9A on the mitochondrial membrane in both wild-type and *Atg*7^−/−^ leukemia cells. When the leukemia cells were treated with the alternative autophagy inhibitor brefeldin A or when the RAB9A was knocked down, this mitophagy was prohibited. This was accompanied by elevated ROS levels and apoptosis as well as reduced DNA damage repair. Therefore, the results suggest that erythroleukemia K562 cells possess an ATG7-independent alternative mitophagic mechanism that functions even when the canonical autophagic process is impaired, thereby maintaining the ability to respond to stresses such as excessive ROS and DNA damage.

Autophagy is a conserved metabolic mechanism that protects cells by delivering potentially toxic macromolecular aggregates (mostly harmful proteins) and damaged or excessive organelles to lysosomes for degradation^1^,^2^. Deletion of *Ulk*1, an essential regulatory gene in the early mammalian autophagic response, results in the accumulation of reticulocytes and a reduction in mature red blood cells. In addition to an impaired clearance of mitochondria, the erythroid cells in *Ulk*1-deleted mice also have impaired clearance of RNA-bound ribosomes^3^. Similarly, lethally irradiated wild-type recipients of *Atg*7-deleted fetal liver transplants have delayed mitochondrial clearance in erythroid cells and deceased red blood cell counts^4^. Moreover, the selective deletion of the *Atg*7 gene in the hematopoietic system results in the accumulation of damaged mitochondria, which is associated with premature cell death. This defect in canonical autophagy caused by *Atg*7 deletion in hematopoietic system causes a multilineage differentiation blockade, resulting in anemia and ultimately leads to the animal’s death^5^,^6^. Therefore, ATG7-dependent canonical autophagy is truly essential in removing damaged or excessive organelles in hematopoietic stem and progenitor cells during the progression of hematopoiesis in mammals.

Leukemia cells are primarily malignantly transformed from hematopoietic stem and progenitor cells during the course of exposure to oncogens, such as ionizing radiation, presumably when rescuing mechanisms, such as autophagy, are inadequate. Like other cancer cells, leukemia cells avoid immune surveillance, bypass signals for apoptosis and necrosis and develop an adaptive metabolism to maintain uncontrollable survival and fast oncogenic growth. Unlike normal cells that produce energy primarily through the oxidation of pyruvate in the mitochondria, oncogenic cells have been reported to predominantly produce energy via enhanced glycolysis in the cytosol even under aerobic conditions^7^. An increasing number of studies show that most malignant cells use glycolysis as a means of energy production regardless of whether they are under normoxic or hypoxic conditions^8^,^9^. These metabolic alterations reflect an adaptive ability for malignant cells to generate ATP.

We have recently reported that, unlike the somatic cells of the mammalian hematopoietic system, hematopoietic stem and progenitor cells are only equipped with canonical autophagy and lack any alternative autophagic machinery^10^. In contrast to normal hematopoietic stem and progenitor cells, our present study demonstrates that erythroleukemia K562 cells acquire an ability for an alternative mechanism of mitophagy. This acquired ability enables the leukemia cells to effectively remove damaged or excessive mitochondria and allows for enhanced reduction of ROS and inhibition of apoptosis. This ultimately increases the competitiveness of these cells for survival and growth over that of hematopoietic cells with normal differentiation potential.

## Results

### *Atg7* deletion in K562 cell line

**Figure 1:**
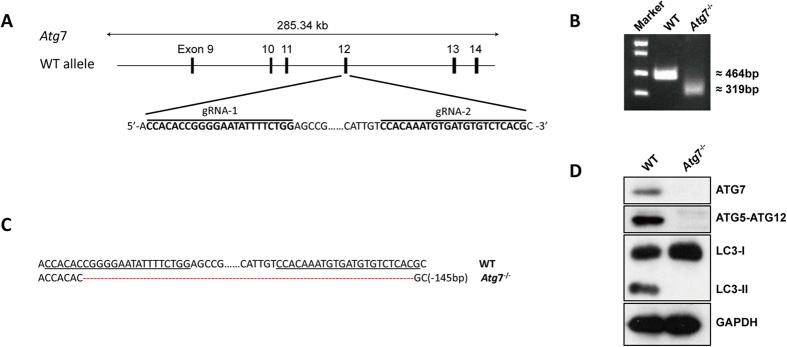
Generation of *Atg*7 knockout K562 cell line with CRISPR/Cas9. (**A**) Schematic diagram of *Atg*7 gene coding region and the targeting loci of gRNA: CRISPR/Cas9. (**B**) DNA electrophoresis of *Atg*7 PCR products in wild-type and *Atg*7^−/−^ K562 cells. (**C**) The DNA sequencing results of wild-type and *Atg*7^−/−^ K562 cells. (**D**) Detection of ATG7, ATG5-ATG12 conjugation and the conversion of LC3-I to LC3-II by immunoblotting.

To explore the possible implications of alternative autophagy in human leukemic cells, we first knocked out a canonical autophagy-essential gene, *Atg*7, with CRISPR/Cas9 to disrupt the capacity for canonical autophagy in the human K562 erythroleukemia cell line (Fig. 1A). Cas9 plasmids with guide RNA targeting exon 12 of *Atg*7 were transfected into K562 cells and single cell clones were analyzed. Genomic PCR followed by electrophoresis showed a reduced band (Fig. 1B), and DNA sequencing further conformed the 145 bp deletion in *Atg*7^−/−^ cells (Fig. 1C). Western blotting analysis showed that *Atg*7 expression was undetectable in *Atg*7^−/−^ cells (Fig. 1D). The Atg7 protein is essential for canonical autophagy, and it mediates autophagic flux involving ATG5-ATG12 conjugation and the conversion of microtubule-associated protein 1 light chain 3-I (LC3-I) to LC3-II^1^,^2^,^11^,^12^,^13^. The failure of ATG5-ATG12 conjugation and LC3-I/LC3-II conversion further indicated the successful knockout of the *Atg*7 gene in K562 cells (Fig. 1D).

### A*tg7* deletion leads to G2/M arrest and reduces differentiation potential

**Figure 2:**
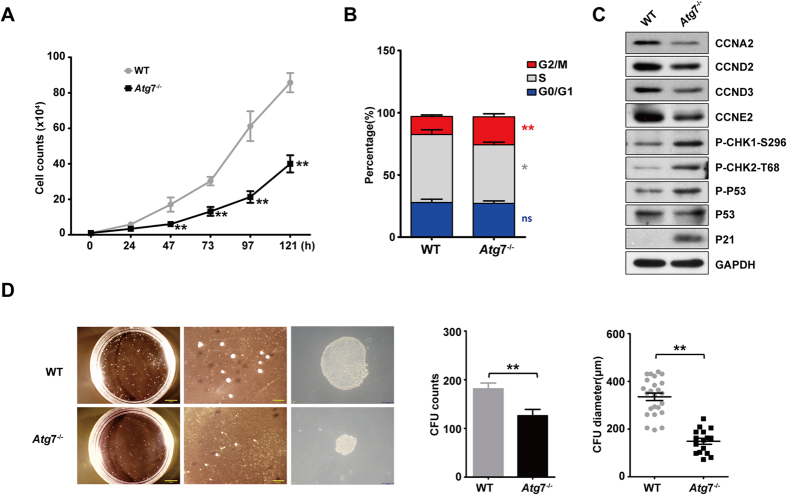
*Atg*7 deletion leads to G2/M arrest and reduced differentiation potential. (**A**) The proliferation curve of wild-type and *Atg*7^−/−^ cells. (**B**) Bar graph represents the DNA histogram of G0/G1, S, G2/M phases of the cell cycle of wild-type and *Atg*7^−/−^ cells. (**C**) Western blotting analysis of cell cycle related proteins (CCNA2, CCND2, CCND3, CCNE1, p53, p-p53, and p21) in wild-type and *Atg*7^−/−^ cells. (**D**) Graphs showing the CFU counts and diameter of wild-type and *Atg*7^−/−^ cells.

To examine the impact of *Atg*7 deletion on leukemia cell proliferation, K562 cells were seeded in plates and cell numbers were counted at the indicated time. The *Atg*7^−/−^ leukemia cells showed a progressive reduction in cell counts in the culture, and the overall growth curve displayed lower proliferation compared with that of *Atg*7 wild-type controls (Fig. 2A). Consistent with the cell growth data, cell cycle analysis showed a significant increase in *Atg*7^−/−^ cells in the G2/M phase and a slight increase in *Atg*7^−/−^ cells in the S phase (Fig. 2B). To analyze the molecular cause of the retarded growth resulting from the *Atg*7 deletion, cell cycle regulatory proteins were examined by immunoblotting. The results showed that knockout of *Atg*7 resulted in reduced levels of CCNA2, CCND2, CCND3, and CCNE1 and, significantly, an enhanced level of p21. Despite no change in the total p53 protein levels, phosphorylated p53 was increased in *Atg*7^−/−^ cells. Phosphorylated CHK1 and CHK2, two upstream regulators of p53, were also upregulated (Fig. 2C). These data suggest that the *Atg*7 deletion-associated reductions in growth were caused by G2/M arrest, which is attributed to the downregulation of CCNA2, CCND2, CCND3, and CCNE1 and the activation of the CHK1/CHK2-p53-p21 pathway. Examination of the differentiation potential of the cells over 121 hours showed that *Atg*7 deletion decreased leukemia cell clone formation, as demonstrated by the colony formation unit (CFU) count, and the diameters of single clones were significantly decreased compared with those of the wild-type controls (Fig. 2D). Examination of apoptosis in wild-type cells and *Atg*7^−/−^ K562 cells in cultures over the same period of time showed that there was no difference in apoptosis between the two groups (Figure S1A). Therefore, the reduction in colony size and the numbers observed in the CFU assay were a consequence of reduced differential potential but not of apoptosis.

### Mitophagy remains functional when ATG7-dependent canonical autophagy is defective

**Figure 3:**
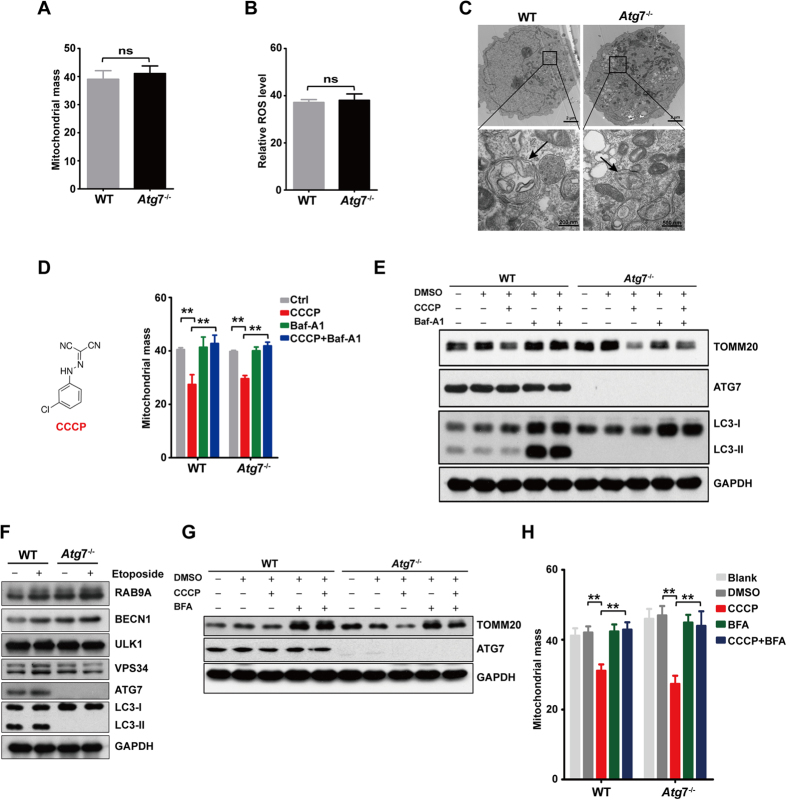
Mitophagy remains functional when ATG7-dependent autophagy is defective. (**A**) Histogram showing the mitochondrial mass by flow cytometry in wild-type and *Atg*7^−/−^ cells. (**B**) A graph showing the ROS level by flow cytometry in wild-type and *Atg*7^−/−^ cells. (**C**) Representative macroautophagy in wild-type and *Atg*7^−/−^ cells. (**D**) Quantification analysis of the mitochondria by flow cytometry in wild-type and *Atg*7^−/−^ cells treated with 20 μM CCCP and 10 nM Baf-A1. (**E**) Detection of the expression mitochondrial protein TOMM20, ATG7 and the conversion of LC3-I to LC3-II in K562 cells treated by 20 μM CCCP and 10 nM Baf-A1 by immunoblotting. (**F**) Detection of the expression of key alternative autophagy related proteins (RAB9A, BECN1, ULK1 and VPS34) by immunoblotting in K562 cells treated with 20 μm Etoposide for 18 h. (**G**) Detection of the expression mitochondrial protein TOMM20, ATG7 and the conversion of LC3-I to LC3-II in wild-type and *Atg*7^−/−^ K562 cells treated by 20 μM CCCP and 0.1 μg/mL BFA by immunoblotting. (**H**) The mitochondrial mass analysis in wild-type and *Atg*7^−/−^ K562 cells treated by 20 μM CCCP and 0.1 μg/mL BFA.

The reduced cell growth caused by the *Atg*7 deletion prompted us to examine whether this defect in canonical autophagy causes increased intracellular stresses such as an increase in ROS. Unexpectedly, the results show that *Atg*7 deletion did not alter mitochondrial mass or ROS levels in K562 leukemia cells (Fig. 3A,B), suggesting that canonical autophagy-defective leukemia cells possibly retained an ability to controlling cellular ROS levels.

During the induction of mammalian autophagy, protein kinase ULK1 is recruited to autophagosome assembly site to bind to other autophagy protein^1^,^2^,^3^. Our result showed that in contrast to non-starvation condition, serum starvation caused punctuate distribution of ULK1 in the cytoplasm in both wild-type and *Atg*7^−/−^ K562 cells (Figure S1B), which suggests that starvation induces the localization of ULK1 onto autophagosome assembly site. Carbonyl cyanide m-chlorophenylhydrazone (CCCP) is a lipid-soluble acid that can lead to mitochondrial depolarization and subsequently induces mitophagy to degrade the damaged mitochondria^14^. After challenge with 20 μM CCCP, ULK1 was aggregated in cytoplasm and colocalized with mitochondria both in wild-type and *Atg*7^−/−^ cells (Figure S1C). This result indicates that CCCP is able to induce mitophagy in both wild-type and *Atg*7^−/−^ K562 cells, and K562 leukemia cells acquires an alternative mitophagy that is independent on ATG7 protein.

Electronic microscopy also indicated an autophagic response in both starved wild-type and *Atg*7-deleted K562 cells, as demonstrated by the formation of autophagosomes, a double-membraned structure enclosing cellular or macromolecular components, and by the formation of autolysosomes, which are formed from the fusion between autophagosomes and lysosomes and appear to be more electron dense under EM (Fig. 3C). These results further indicate that an alternative autophagic mechanism is active in the canonical autophagy-defective leukemia cells, and this alternative autophagy may be responsible for controlling cellular ROS levels.

Mitochondria are a major source for the generation of cellular ROS, and mitochondrial autophagy, known as mitophagy, is a principal mechanism for reducing ROS generation in the cell. We thus measured mitochondrial mass in *Atg*7-deleted cells under stressful conditions. After an 18-h treatment with CCCP, mitochondrial mass declined in both wild-type and *Atg*7^−/−^ leukemia cells, and this degradation could be blocked by 10 nM bafilomycin A1 (Baf-A1), a lysosomal inhibitor (Fig. 3D). The results of flow cytometry were confirmed by western blotting analysis using TOMM20, a marker of mitochondria membrane. Notably, CCCP induced the mitophagic degradation of TOMM20, but brefeldin A (BFA), a specific inhibitor of alternative autophagic degradation^15^, was able to reverse this degradation in both wild-type and *Atg*7^−/−^ K562 cells (Fig. 3E). These results suggest that mitochondria damaged by CCCP were removed by an ATG7-independent alternative autophagic mechanism in the leukemic cells.

To investigate the underlying mechanism by which mitochondria is degraded in *Atg*7^−/−^ leukemia cells, we checked for several key regulatory proteins required in alternative autophagy by western blotting analysis. The results showed that RAB9A and BECN1 were elevated when *Atg*7 was deleted, further suggesting an activation of alternative autophagy. It has been established that 20 μM of etoposide is sufficient to activate alternative autophagy^15^. Indeed, enhanced alternative autophagy was stimulated by 20 μM etoposide, as demonstrated by a further pronounced increase in RAB9A and BECN1 in K562 leukemia cells (Fig. 3F). Western blotting and flow cytometry results showed that 0.1 μg/ml BFA could block the CCCP-induced degradation of TOMM20 that reflects mitochondrial degradation. These results confirmed that CCCP-induced mitophagy occurred in an ATG7-independent manner and that it was blocked by the alternative autophagy inhibitor BFA (Fig. 3G). Flow cytometric analysis of mitochondrial mass also confirmed an inducible mitophagy in *Atg*7-deleted K562 leukemia cells (Fig. 3H).

### Alternative mitophagy is RAB9A-dependent

**Figure 4:**
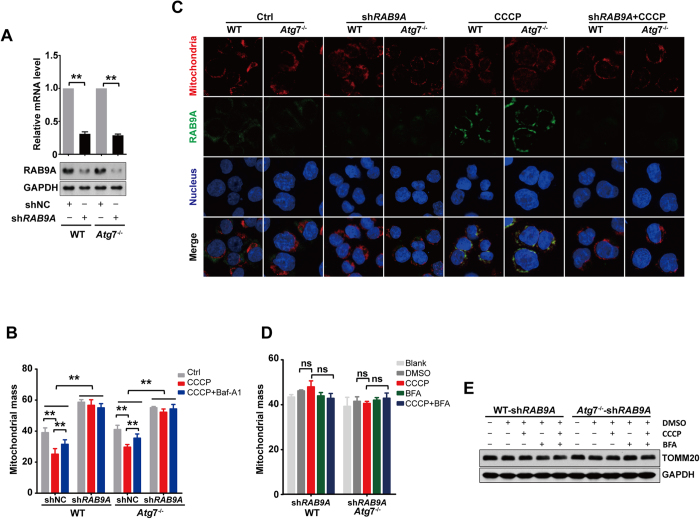
The alternative mitophagy is RAB9A-dependent. (**A**) Detection of the mRNA level of *Rab*9A by quantitative PCR in K562 cells. (**B**) Western blot analysis of RAB9A. (**C**) The colocalization of RAB9A and mitochondria in wild-type and *Atg*7^−/−^ K562 cells treated with CCCP. The nuclei, RAB9A and mitochondria were respectively stained with DAPI (blue), Dylight 488 (green) and Deep Red (red). (**D**) The mitochondrial mass analysis in wild-type and *Atg*7^−/−^ K562 ells treated with CCCP and Baf-A1 when *Rab*9A was knocked down. (**E**) The mitochondrial mass analysis in wild-type and *Atg*7^−/−^ K562 cells treated with CCCP and BFA when *Rab*9A was knocked down. (**F**) Detection of the expression of mitochondrial protein TOMM20 by immunoblotting in wild-type and *Atg*7^−/−^ K562 cells treated with CCCP and BFA when *Rab*9A was knocked down.

As the upregulation of the alternative autophagy-essential protein RAB9A was associated with *Atg*7 deletion in K562 cells, we silenced *Rab*9A with RNA interference to analyze the importance of the RAB9A protein in alternative mitophagy in leukemia cells. Specifically, wild-type and *Atg*7^−/−^ K562 cells were transfected with a lentivirus carrying shRNA against *Rab*9A. As shown by RT-PCR and western blotting results, *Rab*9A was successfully knocked down in both the wild-type and *Atg*7^−/−^ K562 cells (Fig. 4A). *Rab*9A silencing was able to significantly suppress the CCCP-induced alternative degradation of mitochondria in both the wild-type and *Atg*7^−/−^ K562, as observed in the mitochondria mass result obtained with flow cytometry (Fig. 4B). Confocal microscopy showed that RAB9A rarely colocalized with mitochondria under normal conditions, but CCCP treatment increased this colocalization in both the wild-type and *Atg*7^−/−^ K562 cells (Fig. 4C, and Figure S1D), suggesting that CCCP-induced alternative mitophagy depends on RAB9A. As expected, when *Rab*9A was disrupted by RNA interference, CCCP was no longer able to induce the colocalization of RAB9A on mitochondria, suggesting a failure in the activation of alternative mitophagy (Fig. 4C). In support of this observation, the alternative autophagy inhibitor BFA also no longer inhibited mitochondrial autophagy, as determined by cytometric measurements of mitochondrial mass (Fig. 4D). Western blotting analysis also showed that when RAB9A was removed via gene silencing, the mitophagy marker TOMM20 did not change upon treatment with the mitophagy inducer CCCP (Fig. 4E), further suggesting that alternative mitophagy depends on RAB9A. TOMM20 is located in the membrane of mitochondria and thus can also be used for monitoring mitochondrial mass. Therefore, we exposed wild-type and *Atg*7^−/−^ K562 leukemia cells to 3 Gy of nuclear radiation and observed the reduction of TOMM20 in both cell types in response to irradiation. The knockdown of RAB9A by small RNA interference inhibited the reduction of TOMM20 caused by the irradiation (Figure S1E).

Oligomycin and antimycin A in combination are able to induce mitophagy^16^. Consistent with that treated with CCCP, combinational treatment with oligomycin and antimycin A caused a reduction in mitochondrial membrane protein TOMM20, but treatment with Baf-A1 or depletion of RAB9A by small interference RNA inhibited this reduction in both wild-type and *Atg*7^−/−^ cells (Figure S1F). We also examined succinate dehydrogenase (SDHA), a protein located in the matrix of mitochondria. The result showed that similar to TOMM20, SDHA was reduced when wild-type and *Atg*7^−/−^ K562 cells were treated with oligomycin and antimycin A in combination, but treatment with Baf-A1 or depletion of RAB9A by shRNA prevented this reduction in both wild-type and *Atg*7^−/−^ K562 cells (Figure S1F). These results support the idea that *Atg*7^−/−^ K562 cells retain the capacity for alternative mitophagy when ATG7-dependent autophagy is disrupted and that blocking RAB9A prevents alternative mitophagy and results in accumulated mitochondrial mass.

In addition, the induced reduction of mitochondrial mass by combinational treatment with oligomycin and antimycin A was prevented by Baf-A1 in both-wild type and *Atg*7^−/−^ K562 cells, again suggesting a blockade of mitophagy by Baf-A1. Similarly, depletion of RAB9A by shRNA disabled the pharmacologically induced reduction of mitochondrial mass in both wild-type and *Atg7*^−/−^ cells (Figure S1G). This result further supports our notion that K562 leukemia cells acquire an alternative mitophagy capability.

Taken together, these above results suggest that the *Atg*7-deleted leukemia K562 cells retain a mechanism for alternative autophagy that is responsible for the clearance of elevated mitochondria and that the mechanism of alternative mitophagy depends on RAB9A.

### Alternative mitophagy is essential for ROS reduction, anti-apoptosis and DNA damage repair response when canonical autophagy is abolished

**Figure 5:**
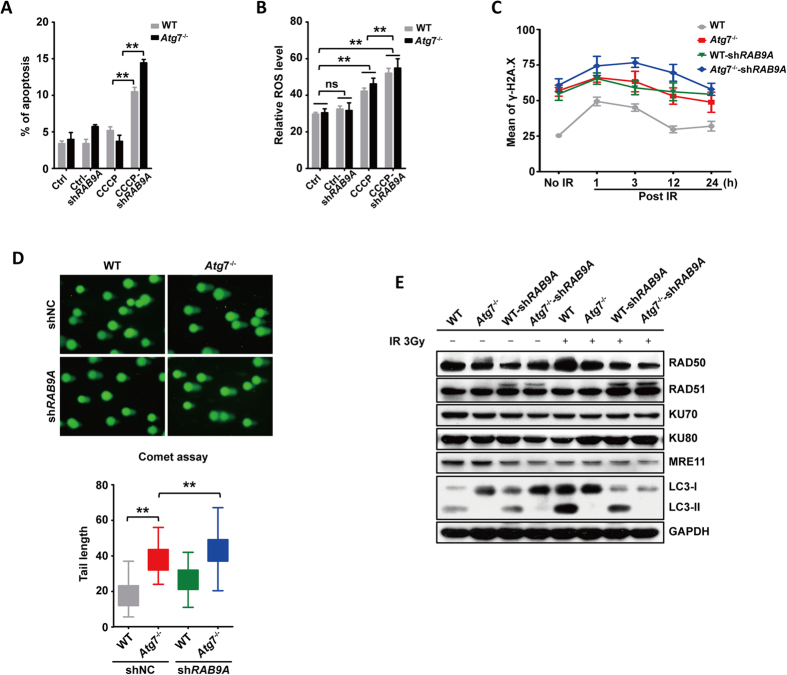
The RAB9A-dependent alternative mitophagy is essential for buffering cellular stresses when canonical autophagy is abolished. (**A**) Quantification analysis of the apoptosis rate in wild-type and *Atg*7^−/−^ K562 cells treated with CCCP when *Rab*9A was knocked down. (**B**) Quantification analysis of the ROS level in wild-type and *Atg*7^−/−^ K562 cells treated with CCCP when *Rab*9A was knocked down. (**C**) The expression of γ-H2AX detected by flow cytometry in wild-type, *Atg*7^−/−^ and *Atg*7^−/−^-sh*Rab*9A K562 cells post 3 Gy ionizing radiation. (**D**) Comet assay of wild-type, *Atg*7^−/−^, wild-type sh*Rab*9A and *Atg*7^−/−^-sh*Rab*9A cells 1 h post 3 Gy ionizing radiation and quantification analysis of the tail length of comet assay of wild-type, *Atg*7^−/−^, wild-type sh*Rab*9A and *Atg*7^−/−^-sh*Rab*9A cells 3h post 3Gy ionizing radiation. (**E**) Detection of the expression of DNA damage repair related proteins (RAD50, RAD51, KU70, KU80, MRE11) and the conversion of LC3-I to LC3-II by immunoblotting in K562 cells post 3 Gy ionizing radiation.

To understand the role of alternative autophagy in the protection of leukemia cells from external and internal stresses, we challenged K562 cells with CCCP. We found that after 18 h of co-culture with CCCP, the apoptosis rate was significantly elevated when *Rab*9A was knocked down in both the wild-type and *Atg*7-knockout cells. Interestingly, compared with that of the wild-type sh*Rab*9A, the apoptosis rate was further increased in the *Atg*7 deleted-sh*RAB*9A cells (Fig. 5A), suggesting an important role for RAB9A-dependent alternative mitophagy in suppressing apoptotic cell death. Similarly, the ROS levels were increased in both the wild-type and *Atg*7^−/−^ K562 cells, and this increase was further enhanced when *Rab*9A was knocked down (Fig. 5B), suggesting that RAB9A-dependent alternative mitophagy also contributed to the reduction of ROS.

To explore whether this alternative mitophagy is implicated in other stress responses, such as DNA damage repair, a 3-Gy nuclear radiation challenge was used to induce DNA breaks, and the expression of γ-H2AX was measured 1 h, 3 h, 12 h and 24 h post-irradiation (IR). The results showed that the mean γ-H2AX expression levels were increased in *Atg*7^−/−^ cells compared with those of wild-type controls under normal conditions. After IR, there was a peak in the mean γ-H2AX expression level at 1 h post-IR. The mean γ-H2AX expression level declined over time after 1 h, and the value returned to a level close to the initial level at 24 h post-IR (Fig. 5C). These data indicate that the DNA damage was repaired at 24 h post-IR in the leukemia cells and that the recovery was slower when *Rab*9A was knocked down in *Atg*7^−/−^ cells. Next, a comet assay was used to visually and statistically examine the level of DNA damage. Similar to the expression of γ-H2AX, *Atg*7^−/−^ K562 cells showed longer tail lengths, compared with those of the wild-type controls, and they had the longest tail lengths at 1 h post-IR when *Rab*9A was knocked down (Fig. 5D). These results indicate that, similar to ATG7-dependent canonical autophagy, alternative mitophagy also plays a role in DNA damage repair. Further examination of DNA damage repair-related proteins revealed that RAD50, a key repair protein in DNA repair, was decreased in *Atg*7^−/−^ cells compared with wild-type controls, and knockdown of *Rab*9A further decreased RAD50 level with or without 3 h of radiation exposure (Fig. 5E). These data suggest that RAB9A is required for the expression or maintenance of RAD50 at a relatively high level and that RAB9A-dependent alternative autophagy is important for DNA damage repair responses when canonical autophagy is abolished in leukemia cells.

## Discussion

We have recently reported that normal hematopoietic stem and progenitor cells in mammals are equipped with one set of autophagic machinery, ATG7-dependent canonical autophagy^10^. Loss of the canonical autophagic mechanism disturbs the maintenance of hematopoietic stem cells^4^,^6^,^10^. We also found that canonical autophagy regulates the quiescence and self-renewal of hematopoietic stem and progenitor cells through regulating cyclin D3, which controls cell cycle entry and the G1/S transition^17^. Furthermore, we reported that canonical autophagy also regulates hematopoiesis via the direct targeting of intracellular Notch^18^. In addition, our recent study indicates that successful autophagic regulation of megakaryopoiesis, megakaryocyte differentiation, and thrombopoiesis in lineage-restricted hematopoietic progenitor cells depends on the efficient clearance of mitochondria^19^. In the present study, we show that in addition to having a canonical autophagic mechanism, erythroleukemia cells possess an ability for alternative mitophagy. This alternative mitophagy is functional regardless of whether their canonical autophagic processes are intact or disrupted. This acquired ability enables the leukemia cells to be more effective than normal hematopoietic stem and progenitors in buffering cellular stresses, particularly in controlling ROS level, which is critical for inhibiting DNA damage and apoptotic cell death.

ROS can function as signaling molecules in various cell types^20^,^21^,^22^,^23^ and can modulate the activation of signal transduction pathways involved in cellular proliferation and differentiation^24^,^25^,^26^. In particular, mitochondrial ROS acts as a progressive trigger for the activation of autophagy, which in turn targets intracellular Notch for degradation to sustain the progression of hematopoietic multilineage differentiation^18^,^27^. However, excessive generation of ROS is destructive in hematopoiesis. ROS-low hematopoietic stem cells have a higher self-renewal potential than ROS-high hematopoietic HSCs^28^, whereas the loss of the ataxia telangiectasia mutated protein or the FoxO transcription factor correlates with higher ROS levels and limits the self-renewal capacity of hematopoietic stem cells in serial transplantations^29^,^30^. These studies suggest that more competitive cells may be equipped with or may acquire more efficient ROS scavenging or reduction mechanisms. Indeed, leukemia stem cells, which show superior growth relative to hematopoietic stem and progenitor cells, acquire an ability to limit ROS. For instance, the expression of the ROS scavenger glutathione peroxidase 3 (GPx3) positively correlates with the frequency of leukemia stem cells in Hoxa9^+^ Meis1-induced leukemia^31^. Similarly, a low ROS level in breast cancer stem cells is associated with an increased expression of *Foxo*1 and the glutathione biosynthesis genes *Gss* and *Gclm*^32^.

Unlike the ATG7-dependent canonical autophagy, which is identified by lipidation and the processing of microtubule-associated protein light chain 3 (LC3) to form LC3-II^1^,^2^, an ATG5/ATG7-independent alternative autophagic mechanism in mouse embryonic fibroblasts was previously reported^15^. Unlike canonical autophagy, autophagosomes in alternative autophagy are generated in a RAB9-dependent manner via the fusion of isolation membranes with vesicles of trans-Golgi and late-endosomal derivation^15^. Our recent study indicates that *Atg*7 deletion leads to an irreversible loss of autophagy in hematopoietic stem and progenitor cells that rely solely on ATG7-dependent canonical autophagy, whereas the same *Atg*7 deletion in myeloid cells triggers an alternative autophagic pathway^10^.

The K562 erythroleukemia cell line was transformed from differentiation-blocked erythroid progenitors. The normal lineage-restricted progenitors depend on ATG7-mediated canonical autophagy for the clearance of mitochondria during generation of mature blood cells because impairment of the canonical autophagic process prevents the erythroid cells from removing mitochondria^5^. Therefore, ATG7-dependent canonical autophagy is the sole autophagic mechanism in the progenitors of red blood cells.

However, in the present study, we observed that there was no difference in mitochondrial mass, ROS generation, DNA damage or apoptosis levels in *Atg*7^−/−^ erythroleukemia cells when compared with wild-type leukemia control cells. Our results indicate that the erythroleukemia K562 cells are armed with both canonical autophagic and alternative mitophagic mechanisms. Even when ATG7-dependent canonical autophagy is dysfunctional, alternative mitophagy is still able to effectively remove damaged or excessive mitochondria to limit ROS production, DNA damage and apoptotic cell death in the leukemia cells. Mechanistically, the upregulation and localization of RAB9A on the mitochondrial membrane is an essential step for the initiation of alternative mitophagy. Similar to the effect of the alternative autophagy inhibitor, the loss of RAB9A prohibited this mitophagy and resulted in elevated ROS levels and increased apoptosis as well as reduced DNA damage repair. Therefore, this alternative mitophagy depends on RAB9A. The RAB9A-dependent alternative mitophagy explains, at least in part, a cellular mechanism for the leukemic advantage in unregulated cell survival and malignant growth.

Due to this leukemic advantage, disrupting canonical autophagy as a part of erythroleukemia therapy only results in cell growth arrest and the inhibition of proliferation; however, disrupting alternative mitophagy leads to a rapid accumulation of ROS that effectively triggers apoptotic cell death. Therefore, both canonical and alternative autophagy pathways should be taken into consideration for anti-leukemia interventions. Our results thus provide new insights in relation to anti-erythroleukemia strategies when considering the targeting of autophagic pathways.

## Methods

### Construction of *Atg*7**-sgRNA vector for the CRISPR/Cas9 system

The backbone plasmids CMV-Cas9-EF1a-puromycin/GFP-U6 optimized for cell transfection was obtained from YSY Biotech Company Ltd (Nanjing, China). To construct the double nicking *Atg*7-sgRNA-guided CRISPR/Cas9 plasmids, a pair of oligos (sgRNA1: TATAGCGTGAGACACATCACATTTG, sgRNA2: TATAGCCAGAAAATATTCCCCGGTG) were designed and subcloned into Cas9 backbone. The two resultant plasmids were used to co-transfect K562 cells with lipofectamine 2000 (Life Techonologies, Thermo Fisher Scientific, Waltham, MA, USA).

### Cell lines and reagents

K562 cell line obtained from ATCC (Manassas, VA, USA) were grown in RPMI-1640 medium (Hyclone, GE healthcare, South Logan, Utah, USA) with 10% fetal bovine serum (Gibco, Thermo fisher scientific, Waltham, MA, USA) in 37 °C, 5% CO_2_ incubator. Cells were incubated with 20 μM CCCP (Sigma-Aldrich, St. Louis, MO, USA), 2 nM and 10 nM bafilomycin A1 (Sigma-Aldrich, St. Louis, MO, USA), 0.1 μg/mL BFA (Beyotime, Nantong, China), Etoposide (Sigma-Aldrich, St. Louis, MO, USA), 100 ng/ml oligomycin (Selleck, Houston, TX, USA), 50 μM antimycin A (Santa Cruz, Dallas, TX, USA) in the indicated experiments. All drugs were dissolved in DMSO.

### Cell proliferation and CFU assay

1 × 10^4^ cells were cultured in 24-well plate, then cells were counted at indicated time. After incubation, cells were washed with PBS. The pellets were fixed in 70% ethanol, washed in PBS, resuspended in PBS containing 50 μg/ml PI and 50 μg/ml RNase A. The DNA content of each cell nucleus was determined by flow cytometry. 300 cells were counted mixed with MethoCult medium (StemCell, Vancouver, BC, Canada) and seeded in 3.5 cm dish. Clones were counted and diameter of each clone was measured under microscope after culture for 7 days.

### Western blot analysis

30 μg of protein was resolved by 10% SDS-PAGE and transferred to PVDF membranes. The membranes were blocked with 5% skim milk-TBS-0.1% Tween 20 for 1 h at room temperature. Antibodies against ATG7, ATG5, CCNA2, CCND2, CCND3, CCNE1, P53, p-P53, P21, RAD50, RAD51, KU70, KU80, MRE11 (Cell Signaling Technology, Danvers, MA, USA), RAB9A, BECN1, ULK1, VPS34, TOMM20 (Abcam, Cambridge, MA, USA) and LC3 (Novus Biologicals, Danvers, CO, USA) were applied to probe the membranes, respectively. The membranes were then washed five times in TBST and incubated with HRP-conjugated secondary antibodies (anti-mouse or anti-rabbit, Cell Signal Technology, USA) diluted 1:2,000 in TBST for 1 h. After 5 times washes, the membranes were developed using an ECL kit (Biological Industries, Kibbutz Beit-Haemek, Israel).

### Confocal microscopy and electron microscopy

For confocal microscopy, 100 nM Mitotracker Deep Red (Life technologies, Thermo fisher scientific, Waltham, MA, USA) was used to stain mitochondria in RPMI 1640 for 30 min at 37 °C, then cells were washed and fixed by 4% paraformaldehyde at RT for 10 mins, rinsed three times in PBS for 5 min each time, and blocked with 5% BSA in PBS (with 0.3% triton X-100) for 1 h. The cells were incubated with the primary antibody (RAB9A, 1:200 diluted in 1% BSA solution) overnight at 4 °C and then with FITC-conjugated secondary antibodies (diluted 1:400) for 2h at RT. Excess of unbound antibody was removed at each step by three times wash with PBS. The nuclear was stained with 20 μg/ml Hoechst 33342 (Invitrogen, Thermo fisher scientific, Waltham, MA, USA) at RT for 10 min. The images were obtained by using confocal microscope (Olympus, Tokyo, Japan). For electron microscopy, cells were collected and fixed with 2.5% glutaricdialdehyde in PBS. After dehydration, the fixed cells were embedded in Epon812. Super thin sections were cut and stained with uranyl acetate. Images were captured under JEM-1010 electron microscope (JEOL, Tokyo, Japan) at indicated magnification.

### Lentivirus production and transduction

The GV298 vector carried shRab9A (5′-CCGAGGATAGGTCAGATCATT-3′) together with negative control (shNC) was from GeneChem CO, Ltd (Shanghai, China). Package plasmids PSPA and PMO-2G were kindly from Dr. Sudan He (Soochow University, Suzhou, China). The viral production was performed with standard protocol. In brief, 5 μg target plasmid, 3.2 μg PSPA and 1.8 μg PMO-2G were co-transfected into 293T cells with calcium precipitate method. Virus containing media (VCM) was collected 48 hours after transfection. 1 × 10^4^ wild type and ATG7 KO cells were mixed with 500 μL VCM overnight, and then the cells were washed with RPMI 1640 for 3 times. 48 hours later, mCherry^+ ^ cells were sorted and collected for further analysis.

### Flow cytometry

After indicated treatment, cells were stained with 100 nM Mitotracker Deep Red in RPMI 1640 for 30 min at 37 °C, then the mean fluorescence intensity (MFI) was detected by flow cytometry. Differences in mitochondrial mass were analyzed using an unpaired *t*-test. Cellular reactive oxygen species were measured with Cello (Life technologies, Thermo fisher scientific, Waltham, MA, USA) reagent in the same way. After ionizing radiation, cells were washed and fixed by 4% paraformaldehyde at RT for 10 mins, washed by PBS, and then resuspended in 0.3% triton X-100 for 30 min at 4 °C. The cells were incubated with phospho-H2AX (S139) antibody (eBioscience, San Diego, CA, USA) for 1 h at 4 °C. The MFI was detected by flow cytometry. Differences were analyzed using an unpaired *t*-test.

### Comet assay

The comet assay was performed using the Comet Assay kit (Trevigen, Gaithersburg, MD, USA) following the manufacturer’s instructions. Briefly, after ionizing radiation, cells were fixed on the slides and stained with SYBR Green, then observed by fluorescence microscopy with 490-nm filter. The tail length was measured by software (Olympus, Tokyo, Japan), differences were analyzed using an unpaired *t*-test.

## Additional Information

**How to cite this article**: Wang, J. *et al*. Erythroleukemia cells acquire an alternative mitophagy capability. *Sci. Rep.* **6**, 24641; doi: 10.1038/srep24641 (2016).

## Supplementary Material

Supplementary Information
