# Supplementary material for: Erythroleukemia cells acquire an alternative mitophagy capability
Source: Sci Rep. 2016 Apr 19;6:24641. doi: 10.1038/srep24641 (PMC4835698; doi:10.1038/srep24641)
Supplement: Supplementary Information [file srep24641-s1.pdf]

Erythroleukemia cells acquire an alternative mitophagy capability

Jian Wang*, Yixuan Fang*, Lili Yan, Na Yuan, Suping Zhang, Li Xu, Meilan Nie, Xiaoying Zhang, Jianrong Wang

Hematology Center of Cyrus Tang Medical Institute, Jiangsu Institute of Hematology, Collaborative Innovation Center of Hematology, Jiangsu Key Laboratory for Stem Cell Research, Soochow University School of Medicine, Suzhou 215123, China.

Correspondence and requests for materials should be addressed to: Jianrong Wang (jrwang@suda.edu.cn). *These authors contributed equally to this work.

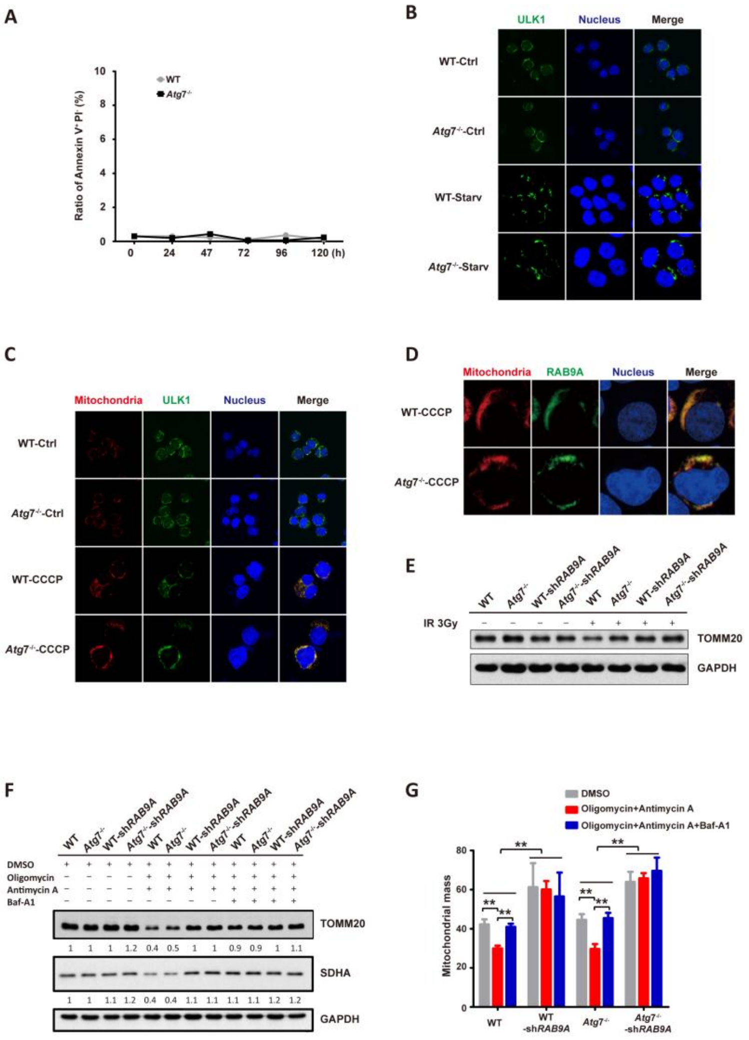

Supplemental Figure 1. Erythroleukemia cells acquire an alternative mitophagy capability. (**A**) Quantification analysis of the apoptosis level in wild-type and *Atg*7$^{-/-}$ K562 cells with indicated time. (**B)** The localization of ULK1 in K562 cells under normal and serum deprivation in wild-type and *Atg*7$^{-/-}$ K562 cells. The ULK1 was stained with Dylight 488 (green). (**C)** The colocalization of ULK1 and mitochondria in wild-type and *Atg*7$^{-/-}$ K562 cells treated with CCCP. The ULK1 was stained with Dylight 488 (green) and mitochondria was stained with Deep Red (red). (**D**) The colocalization of RAB9A and mitochondria in wild-type and *Atg*7$^{-/-}$ K562 cells treated with CCCP. RAB9A was stained with Dylight 488 (green) and mitochondria was stained with Deep Red (red). (**E**) Detection of the expression of TOMM20 by immunoblotting in wild-type and *Atg*7$^{-/-}$ K562 cells post 3Gy ionizing radiation. (**F**) Detection of the expression of SDHA and TOMM20 by immunoblotting in wild-type and *Atg*7$^{-/-}$ K562 cells with indicated drugs. The expression levels were quantified with number indicated. (**G**) Quantification analysis of mitochondrial mass in wild-type and *Atg*7$^{-/-}$ K652 cells when cultured with oligomycin (100ng/ml), antimycin A (50µM) and Baf-A1 (10nM).
